# 2-(1,3-Benzothia­zol-2-ylimino­meth­yl)-2-naphthol

**DOI:** 10.1107/S1600536809008514

**Published:** 2009-03-14

**Authors:** Khadija O. Badahdaha, Abdullah Mohamed Asiri, Seik Weng Ng

**Affiliations:** a Chemistry Department, Faculty of Science, King Abdul Aziz University, Jeddah, Saudi Arabia; b Department of Chemistry, University of Malaya, 50603 Kuala Lumpur, Malaysia

## Abstract

In the title mol­ecule, C_18_H_12_N_2_OS, the dihedral angle between the two fused-ring systems is 7.2 (1)°. The hydr­oxy group forms an intra­molecular hydrogen bond with the imino group.

## Related literature

For the crystal structures of other Schiff bases derived by condensing benzthia­zolyl-2-amine with aldehydes/ketones, see: Büyükgüngör *et al.* (2004 ▶); Cannon *et al.* (2001 ▶); Guo *et al.* (2002 ▶); Saraçoğlu *et al.* (2004 ▶).
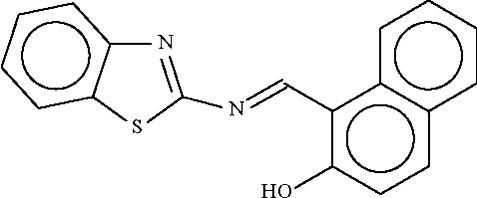


## Experimental

### 

#### Crystal data

C_18_H_12_N_2_OS
*M* *_r_* = 304.36
Monoclinic, 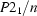

*a* = 9.6398 (2) Å
*b* = 14.9687 (4) Å
*c* = 9.6646 (2) Å
β = 101.323 (2)°
*V* = 1367.41 (5) Å^3^
*Z* = 4
Mo *K*α radiation
μ = 0.24 mm^−1^
*T* = 123 K
0.25 × 0.20 × 0.05 mm

#### Data collection

Bruker SMART APEX diffractometer
Absorption correction: multi-scan (*SADABS*; Sheldrick, 1996 ▶) *T*_min_ = 0.943, *T*_max_ = 0.988
12442 measured reflections
3139 independent reflections
2396 reflections with *I* > 2σ(*I*)
*R*_int_ = 0.051

#### Refinement

*R*[*F* ^2^ > 2σ(*F* ^2^)] = 0.043
*wR*(*F* ^2^) = 0.122
*S* = 1.02
3139 reflections
203 parameters
1 restraint
H atoms treated by a mixture of independent and constrained refinement
Δρ_max_ = 0.53 e Å^−3^
Δρ_min_ = −0.23 e Å^−3^

### 

Data collection: *APEX2* (Bruker, 2008 ▶); cell refinement: *SAINT* (Bruker, 2008 ▶); data reduction: *SAINT*; program(s) used to solve structure: *SHELXS97* (Sheldrick, 2008 ▶); program(s) used to refine structure: *SHELXL97* (Sheldrick, 2008 ▶); molecular graphics: *X-SEED* (Barbour, 2001 ▶); software used to prepare material for publication: *publCIF* (Westrip, 2009 ▶).

## Supplementary Material

Crystal structure: contains datablocks global, I. DOI: 10.1107/S1600536809008514/lh2783sup1.cif

Structure factors: contains datablocks I. DOI: 10.1107/S1600536809008514/lh2783Isup2.hkl

Additional supplementary materials:  crystallographic information; 3D view; checkCIF report

## Figures and Tables

**Table 1:** Hydrogen-bond geometry (Å, °)

*D*—H⋯*A*	*D*—H	H⋯*A*	*D*⋯*A*	*D*—H⋯*A*
O1—H1⋯N1	0.84 (1)	1.82 (2)	2.573 (2)	148 (3)

**Fig. 1.**
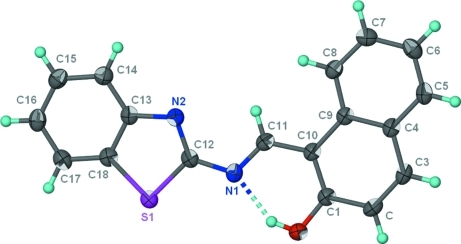
Thermal ellisoid plot (Barbour, 2001) of C18H12N2OS; probability levels are set at 70% and H-atoms are drawn as spheres of arbitrary radius. The dashed line denotes the hydrogen bond.

## supplementary crystallographic information

### Comment

The molecular structure of the title compound is shown in Fig. 1.

### Experimental

2-Aminobenzothiazole (4.0 g, 26.6 mmol) dissolved in ethanol (25 ml) was added to 2-hydroxybenzaldehyde (4.58 g, 26.6 mmol) dissolved in ethanol (25 ml). The mixture was heated for another hour. The solid that separated from the reaction mixture was isolated and recrystallized from ethanol.

### Refinement

Carbon-bound H-atoms were placed in calculated positions (C–H 0.95 Å) and were included in the refinement in the riding model approximation, with *U*(H) fixed at 1.2*U*(C).

The hydroxy H-atom was located in a difference Fouier map, and was refined with a distance restraint of O–H 0.84±0.01 Å; its isotropic displacement parameter was refined.

### Crystal data

**Table:**

C_18_H_12_N_2_OS	*F*(000) = 632
*M**_r_* = 304.36	*D*_x_ = 1.481 Mg m^−^^3^
Monoclinic, *P*2_1_/*n*	Mo *K*α radiation, λ = 0.71073 Å
Hall symbol: -P 2yn	Cell parameters from 2677 reflections
*a* = 9.6398 (2) Å	θ = 2.5–28.1°
*b* = 14.9687 (4) Å	µ = 0.24 mm^−^^1^
*c* = 9.6646 (2) Å	*T* = 123 K
β = 101.323 (2)°	Irregular block, orange
*V* = 1367.41 (5) Å^3^	0.25 × 0.20 × 0.05 mm
*Z* = 4	

### Data collection

**Table:**

Bruker SMART APEX diffractometer	3139 independent reflections
Radiation source: fine-focus sealed tube	2396 reflections with *I* > 2˘*I*)
graphite	*R*_int_ = 0.051
ω scans	θ_max_ = 27.5°, θ_min_ = 2.5°
Absorption correction: multi-scan (*SADABS*; Sheldrick, 1996)	*h* = −12→12
*T*_min_ = 0.943, *T*_max_ = 0.988	*k* = −19→19
12442 measured reflections	*l* = −12→12

### Refinement

**Table:**

Refinement on *F*^2^	Primary atom site location: structure-invariant direct methods
Least-squares matrix: full	Secondary atom site location: difference Fourier map
*R*[*F*^2^ > 2σ(*F*^2^)] = 0.043	Hydrogen site location: inferred from neighbouring sites
*wR*(*F*^2^) = 0.122	H atoms treated by a mixture of independent and constrained refinement
*S* = 1.02	*w* = 1/[σ^2^(*F*_o_^2^) + (0.0612*P*)^2^ + 0.543*P*] where *P* = (*F*_o_^2^ + 2*F*_c_^2^)/3
3139 reflections	(Δ/σ)_max_ = 0.001
203 parameters	Δρ_max_ = 0.53 e Å^−^^3^
1 restraint	Δρ_min_ = −0.23 e Å^−^^3^

### Special details

**Table:**

Geometry. All e.s.d.'s (except the e.s.d. in the dihedral angle between two l.s. planes) are estimated using the full covariance matrix. The cell e.s.d.'s are taken into account individually in the estimation of e.s.d.'s in distances, angles and torsion angles; correlations between e.s.d.'s in cell parameters are only used when they are defined by crystal symmetry. An approximate (isotropic) treatment of cell e.s.d.'s is used for estimating e.s.d.'s involving l.s. planes.

### Fractional atomic coordinates and isotropic or equivalent isotropic displacement parameters (Å^2^)

**Table:**

	*x*	*y*	*z*	*U*_iso_*/*U*_eq_	
S1	0.62414 (5)	0.57067 (3)	0.89471 (5)	0.02326 (15)	
O1	0.28598 (15)	0.35583 (9)	0.77536 (15)	0.0261 (3)	
H1	0.348 (2)	0.3964 (14)	0.782 (3)	0.057 (9)*	
N1	0.41330 (16)	0.49518 (10)	0.70645 (17)	0.0214 (4)	
N2	0.53279 (16)	0.62326 (11)	0.63631 (17)	0.0218 (3)	
C1	0.19789 (19)	0.36352 (12)	0.6498 (2)	0.0207 (4)	
C2	0.0903 (2)	0.29840 (13)	0.6173 (2)	0.0236 (4)	
H2	0.0831	0.2524	0.6833	0.028*	
C3	−0.0028 (2)	0.30118 (13)	0.4924 (2)	0.0227 (4)	
H3	−0.0733	0.2562	0.4715	0.027*	
C4	0.00270 (19)	0.37013 (12)	0.3918 (2)	0.0205 (4)	
C5	−0.0966 (2)	0.37268 (13)	0.2635 (2)	0.0242 (4)	
H5	−0.1667	0.3274	0.2434	0.029*	
C6	−0.0935 (2)	0.43936 (14)	0.1678 (2)	0.0257 (4)	
H6	−0.1613	0.4407	0.0819	0.031*	
C7	0.0109 (2)	0.50600 (13)	0.1975 (2)	0.0246 (4)	
H7	0.0131	0.5526	0.1312	0.030*	
C8	0.1093 (2)	0.50481 (13)	0.3206 (2)	0.0219 (4)	
H8	0.1790	0.5504	0.3380	0.026*	
C9	0.10912 (19)	0.43680 (12)	0.42252 (19)	0.0189 (4)	
C10	0.20982 (19)	0.43225 (12)	0.5546 (2)	0.0190 (4)	
C11	0.3197 (2)	0.49736 (13)	0.5896 (2)	0.0210 (4)	
H11	0.3245	0.5443	0.5245	0.025*	
C12	0.51359 (19)	0.56351 (12)	0.7280 (2)	0.0205 (4)	
C13	0.64047 (19)	0.68133 (12)	0.6951 (2)	0.0199 (4)	
C14	0.6849 (2)	0.75430 (13)	0.6250 (2)	0.0230 (4)	
H14	0.6414	0.7675	0.5303	0.028*	
C15	0.7934 (2)	0.80674 (13)	0.6964 (2)	0.0251 (4)	
H15	0.8252	0.8562	0.6495	0.030*	
C16	0.8576 (2)	0.78866 (13)	0.8360 (2)	0.0256 (4)	
H16	0.9320	0.8260	0.8826	0.031*	
C17	0.8139 (2)	0.71684 (13)	0.9077 (2)	0.0245 (4)	
H17	0.8570	0.7046	1.0029	0.029*	
C18	0.7052 (2)	0.66319 (13)	0.8359 (2)	0.0214 (4)	

### Atomic displacement parameters (Å^2^)

**Table:**

	*U*^11^	*U*^22^	*U*^33^	*U*^12^	*U*^13^	*U*^23^
S1	0.0230 (3)	0.0244 (3)	0.0214 (3)	−0.00141 (19)	0.00199 (18)	0.00103 (19)
O1	0.0272 (7)	0.0245 (8)	0.0252 (7)	−0.0027 (6)	0.0017 (6)	0.0047 (6)
N1	0.0198 (8)	0.0208 (8)	0.0237 (8)	−0.0009 (6)	0.0049 (6)	−0.0016 (7)
N2	0.0210 (8)	0.0221 (8)	0.0220 (8)	−0.0008 (6)	0.0034 (6)	−0.0001 (7)
C1	0.0204 (9)	0.0202 (9)	0.0224 (10)	0.0019 (7)	0.0065 (7)	−0.0006 (7)
C2	0.0261 (10)	0.0194 (9)	0.0276 (10)	−0.0001 (8)	0.0112 (8)	0.0027 (8)
C3	0.0211 (9)	0.0188 (9)	0.0301 (11)	−0.0030 (7)	0.0098 (8)	−0.0036 (8)
C4	0.0197 (9)	0.0193 (9)	0.0241 (10)	0.0010 (7)	0.0081 (7)	−0.0031 (8)
C5	0.0198 (9)	0.0231 (10)	0.0295 (11)	−0.0015 (8)	0.0045 (8)	−0.0066 (8)
C6	0.0240 (10)	0.0278 (11)	0.0235 (10)	0.0055 (8)	0.0006 (8)	−0.0047 (8)
C7	0.0280 (10)	0.0238 (10)	0.0230 (10)	0.0048 (8)	0.0074 (8)	0.0007 (8)
C8	0.0227 (9)	0.0194 (9)	0.0249 (10)	−0.0009 (7)	0.0078 (8)	−0.0013 (8)
C9	0.0185 (9)	0.0178 (9)	0.0216 (9)	0.0025 (7)	0.0067 (7)	−0.0019 (7)
C10	0.0183 (9)	0.0176 (9)	0.0223 (9)	0.0018 (7)	0.0072 (7)	−0.0015 (7)
C11	0.0218 (9)	0.0192 (9)	0.0236 (10)	0.0007 (7)	0.0082 (7)	0.0005 (8)
C12	0.0191 (9)	0.0213 (9)	0.0210 (9)	0.0024 (7)	0.0035 (7)	−0.0024 (7)
C13	0.0181 (9)	0.0186 (9)	0.0236 (10)	0.0031 (7)	0.0053 (7)	−0.0030 (7)
C14	0.0229 (9)	0.0213 (10)	0.0247 (10)	0.0020 (8)	0.0050 (8)	−0.0003 (8)
C15	0.0256 (10)	0.0201 (10)	0.0311 (11)	0.0003 (8)	0.0093 (8)	−0.0006 (8)
C16	0.0214 (9)	0.0235 (10)	0.0319 (11)	−0.0011 (8)	0.0057 (8)	−0.0065 (8)
C17	0.0223 (10)	0.0280 (11)	0.0229 (10)	0.0028 (8)	0.0037 (8)	−0.0041 (8)
C18	0.0204 (9)	0.0220 (9)	0.0227 (10)	0.0028 (8)	0.0067 (7)	−0.0026 (8)

### Geometric parameters (Å, °)

**Table:**

S1—C18	1.740 (2)	C6—H6	0.9500
S1—C12	1.7519 (19)	C7—C8	1.368 (3)
O1—C1	1.343 (2)	C7—H7	0.9500
O1—H1	0.843 (10)	C8—C9	1.417 (3)
N1—C11	1.300 (2)	C8—H8	0.9500
N1—C12	1.395 (2)	C9—C10	1.445 (3)
N2—C12	1.298 (2)	C10—C11	1.430 (3)
N2—C13	1.387 (2)	C11—H11	0.9500
C1—C10	1.400 (3)	C13—C14	1.396 (3)
C1—C2	1.413 (3)	C13—C18	1.408 (3)
C2—C3	1.356 (3)	C14—C15	1.380 (3)
C2—H2	0.9500	C14—H14	0.9500
C3—C4	1.426 (3)	C15—C16	1.397 (3)
C3—H3	0.9500	C15—H15	0.9500
C4—C5	1.410 (3)	C16—C17	1.388 (3)
C4—C9	1.420 (3)	C16—H16	0.9500
C5—C6	1.365 (3)	C17—C18	1.392 (3)
C5—H5	0.9500	C17—H17	0.9500
C6—C7	1.406 (3)		
			
C18—S1—C12	88.83 (9)	C8—C9—C10	123.76 (17)
C1—O1—H1	109 (2)	C4—C9—C10	118.92 (17)
C11—N1—C12	116.99 (16)	C1—C10—C11	119.86 (17)
C12—N2—C13	110.36 (16)	C1—C10—C9	119.17 (17)
O1—C1—C10	122.56 (17)	C11—C10—C9	120.96 (17)
O1—C1—C2	116.63 (17)	N1—C11—C10	122.94 (18)
C10—C1—C2	120.82 (18)	N1—C11—H11	118.5
C3—C2—C1	120.34 (18)	C10—C11—H11	118.5
C3—C2—H2	119.8	N2—C12—N1	126.25 (17)
C1—C2—H2	119.8	N2—C12—S1	116.32 (14)
C2—C3—C4	121.51 (18)	N1—C12—S1	117.44 (14)
C2—C3—H3	119.2	N2—C13—C14	124.58 (17)
C4—C3—H3	119.2	N2—C13—C18	115.42 (17)
C5—C4—C9	120.17 (18)	C14—C13—C18	120.00 (17)
C5—C4—C3	120.62 (17)	C15—C14—C13	118.50 (18)
C9—C4—C3	119.21 (18)	C15—C14—H14	120.8
C6—C5—C4	120.93 (18)	C13—C14—H14	120.7
C6—C5—H5	119.5	C14—C15—C16	121.43 (19)
C4—C5—H5	119.5	C14—C15—H15	119.3
C5—C6—C7	119.37 (19)	C16—C15—H15	119.3
C5—C6—H6	120.3	C17—C16—C15	120.81 (19)
C7—C6—H6	120.3	C17—C16—H16	119.6
C8—C7—C6	120.97 (19)	C15—C16—H16	119.6
C8—C7—H7	119.5	C16—C17—C18	118.07 (19)
C6—C7—H7	119.5	C16—C17—H17	121.0
C7—C8—C9	121.24 (18)	C18—C17—H17	121.0
C7—C8—H8	119.4	C17—C18—C13	121.18 (18)
C9—C8—H8	119.4	C17—C18—S1	129.72 (16)
C8—C9—C4	117.32 (17)	C13—C18—S1	109.07 (14)
			
O1—C1—C2—C3	−179.77 (17)	C12—N1—C11—C10	−179.24 (16)
C10—C1—C2—C3	0.5 (3)	C1—C10—C11—N1	1.6 (3)
C1—C2—C3—C4	−1.3 (3)	C9—C10—C11—N1	−179.23 (17)
C2—C3—C4—C5	−178.90 (18)	C13—N2—C12—N1	179.17 (17)
C2—C3—C4—C9	0.7 (3)	C13—N2—C12—S1	−0.3 (2)
C9—C4—C5—C6	−0.8 (3)	C11—N1—C12—N2	−8.4 (3)
C3—C4—C5—C6	178.85 (18)	C11—N1—C12—S1	171.09 (14)
C4—C5—C6—C7	0.4 (3)	C18—S1—C12—N2	−0.17 (15)
C5—C6—C7—C8	0.2 (3)	C18—S1—C12—N1	−179.72 (15)
C6—C7—C8—C9	−0.3 (3)	C12—N2—C13—C14	−178.50 (17)
C7—C8—C9—C4	−0.1 (3)	C12—N2—C13—C18	0.8 (2)
C7—C8—C9—C10	−179.70 (17)	N2—C13—C14—C15	179.92 (17)
C5—C4—C9—C8	0.6 (3)	C18—C13—C14—C15	0.6 (3)
C3—C4—C9—C8	−179.03 (16)	C13—C14—C15—C16	−0.6 (3)
C5—C4—C9—C10	−179.76 (16)	C14—C15—C16—C17	0.1 (3)
C3—C4—C9—C10	0.6 (3)	C15—C16—C17—C18	0.4 (3)
O1—C1—C10—C11	0.4 (3)	C16—C17—C18—C13	−0.4 (3)
C2—C1—C10—C11	−179.91 (17)	C16—C17—C18—S1	−178.59 (15)
O1—C1—C10—C9	−178.88 (16)	N2—C13—C18—C17	−179.46 (17)
C2—C1—C10—C9	0.9 (3)	C14—C13—C18—C17	−0.1 (3)
C8—C9—C10—C1	178.24 (17)	N2—C13—C18—S1	−0.9 (2)
C4—C9—C10—C1	−1.4 (3)	C14—C13—C18—S1	178.42 (14)
C8—C9—C10—C11	−1.0 (3)	C12—S1—C18—C17	178.94 (19)
C4—C9—C10—C11	179.39 (17)	C12—S1—C18—C13	0.60 (14)

### Hydrogen-bond geometry (Å, °)

**Table:**

*D*—H···*A*	*D*—H	H···*A*	*D*···*A*	*D*—H···*A*
O1—H1···N1	0.84 (1)	1.82 (2)	2.573 (2)	148 (3)
